# Screening antiseizure medications to break the cycle between neuronal hyperexcitability and Alzheimer's disease

**DOI:** 10.1177/13872877251364556

**Published:** 2025-08-03

**Authors:** Aaron J Barbour

**Affiliations:** 1 Department of Neurology, Perelman School of Medicine, University of Pennsylvania, Philadelphia, PA, USA

**Keywords:** Alzheimer's disease, seizures, sex differences

## Abstract

Epilepsy is commonly comorbid with Alzheimer's disease and is now well established to accelerate disease course. In the present study, Knox and colleagues evaluated the efficacy of several antiseizure medications (ASMs) in mitigating seizure induction in two aged Alzheimer's disease mouse models with distinct etiologies. They found differential responses to seizure induction and ASM treatment across sexes and models. These results reveal a complex interplay between sex, Alzheimer's disease risk genes, and neuronal hyperexcitability that suggest a tailored approach to seizure control may maximize therapeutic benefit in Alzheimer's disease.

Alzheimer's disease is characterized by the accumulation and spread of amyloid-β (Aβ) plaques and tau neurofibrillary tangles, which are believed to drive neuronal dysfunction, cell death, and cognitive decline.^1^ However, to date, therapies designed to target these hallmark pathologies have yielded, at best, modest clinical benefits,^2^ underscoring the critical need for novel treatment strategies. Several additional components have emerged as drivers of disease progression, including neuroinflammation,^3^ metabolic dysfunction,^4^ blood-brain barrier disruption,^5^ and neuronal hyperexcitability,^6^ suggesting therapeutic promise in targeting these factors. Seizures and epileptiform activity are common comorbidities in Alzheimer's disease (up to 64%) and worsen cognitive and pathological outcomes.^7–10^ Indeed, there is a complex interplay between neuronal excitability and Alzheimer's disease pathology. While some studies show reduced neuronal activity in specific contexts,^11,12^ a positive feedback loop between neuronal hyperexcitability and Aβ is well accepted, whereby amyloid plaques increase neuronal excitability,^13^ likely contributing to the increased incidence of epileptiform activity in Alzheimer's disease. This heightened excitability, in turn, promotes both plaque and tau accumulation and spread. Notably, experimentally induced hyperactivity and seizures exacerbate Aβ and tau pathology,^8,14,15^ suggesting that targeting this pathological hyperactivity with antiseizure medications (ASMs) may slow disease progression. While there is clear efficacy in controlling seizures with ASMs, the use of these drugs in the context of Alzheimer's disease is underexplored. To address this gap, Knox and colleagues evaluated several common ASMs for their efficacy to ameliorate induced seizures in two aged Alzheimer's disease mouse models.^16^

While EEG is the gold standard for seizure monitoring, these experiments are costly, laborious, and can be lengthy if monitoring infrequent spontaneous seizures, thus limiting throughput. Knox et al.^16^ utilized a corneal seizure kindling model and behavioral evaluation of seizure activity, providing a moderate throughput to screen acute ASM efficacy in Alzheimer's disease mouse models. The authors examined several common ASMs including levetiracetam, valproic acid, lamotrigine, phenobarbital, and gabapentin, for their seizure modifying effects in two aged familial Alzheimer's disease mouse models, one with plaque pathology, the APP/PS1 mouse, and one without, the PSEN2-N141I mouse. Their study identified increased mortality and worsened seizure severity due to seizure kindling in male APP/PS1 mice, further corroborating the impact of plaque pathology on neuronal hyperexcitability. In contrast, they found reduced seizure susceptibility in aged male PSEN2-N141I mice. While contradictory to results garnered in young PSEN2-N141I mice^17^ and other Alzheimer's disease models,^8,18^ these results confirm their prior report^17^ and highlight a complex interplay between sex, disease stage, and underlying etiology on the impacts to network excitability.

Overall, acute ASM treatment generally reduced seizure severity following corneal kindling, but their efficacies varied by model and sex.^16^ In APP/PS1 mice, Levetiracetam was more effective and lamotrigine less effective in males compared to controls, whereas levetiracetam, valproic acid, and gabapentin showed greater efficacy in females.^16^ In male PSEN2-141I, all ASMs demonstrated similar efficacy compared to controls, while in females, lamotrigine and gabapentin were more effective than in controls.^16^ Notably, phenobarbital was effective across all model and control mice, and levetiracetam, valproic acid, and gabapentin showed elevated efficacy in the Alzheimer's disease model mice, highlighting these ASMs as promising candidates for further study. The sex differences identified in these studies may have important clinical implications and could reflect underlying hormonal influences on neuronal excitability, such as estrogen's modulation of synaptic function.^19^ Knox et al. did not evaluate the efficacy of chronic ASM treatments—a critical step in evaluating the long-term therapeutic potential of these drugs. Additionally, behavioral seizure monitoring may miss subtle alterations including non-motor epileptiform activity and changes in spectral power. Overall, these data demonstrate potent, yet heterogenous efficacies of ASMs on seizure control in aged Alzheimer's disease models, indicating their clinical potential and the possible benefits of a tailored approach to seizure control in the Alzheimer's disease population.

In addition to controlling seizures, which may help mitigate cognitive decline, ASMs may also influence pathological progression, as neuronal hyperactivity can increase Aβ and tau levels. Indeed, levetiracetam, lamotrigine, and valproic acid reduced pathology and improved cognition in Alzheimer's disease mouse models.^20–23^ While not examined in the present study, specific ASMs may have a direct effect on the development of pathology. Notably, valproic acid blocks activity from GSK3β,^24^ a tau kinase, further underscoring its potential to slow a critical step in neurofibrillary tangle development. While valproic acid has not had convincing translational success to date,^25^ levetiracetam is currently the focus of clinical trials and has shown cognitive benefits in Alzheimer's disease patients with comorbid epileptiform activity and in patients with mild cognitive impairment.^26,27^ While ASMs offer a promising and accessible approach to modulating neuronal hyperexcitability in Alzheimer's disease, limitations must also be considered. Long-term use of ASMs has been associated with cognitive side effects,^28^ elderly patients are often prescribed multiple medications, further risking drug-drug interactions, and there is potential for off-target effects. These considerations further underscore the need for studies evaluating chronic treatment outcomes in the context of Alzheimer's disease.

While encouraging advancements have been made in targeting Aβ pathology with monoclonal antibodies, these treatments are expensive, the cognitive benefits are limited, and there are major health risks associated with such treatments.^2^ Alternatively, ASMs are largely safe, FDA approved, and widely accessible. Although this may not be a one-size-fits-all approach, as ASM efficacy is variable and likely influenced by biological factors. To move toward a personalized treatment paradigm, future efforts should focus on identifying reliable stratification criteria. Based on the findings from Knox et al.^16^ and clinical studies, biomarkers such as epileptiform activity,^27^ sex hormone levels, genetic risk factors, and disease stage^25,26^ may guide ASM selection and timing for Alzheimer's disease treatment. For future clinical success, a tailored approach to seizure reduction will be critical to maximize ASM efficacy and effectively disrupt the cycle between Alzheimer's disease pathology and neuronal hyperexcitability.
